# Interrelationships between the structural, spectroscopic, and antibacterial properties of nanoscale (< 50 nm) cerium oxides

**DOI:** 10.1038/s41598-021-00222-9

**Published:** 2021-10-22

**Authors:** Neelam Iqbal, Antonios Anastasiou, Zabeada Aslam, El Mostafa Raif, Thuy Do, Peter V. Giannoudis, Animesh Jha

**Affiliations:** 1 School of Chemical and Process Engineering, University of Leeds, Leeds, UK; 2 Department of Chemical Engineering and Analytical Science, University of Manchester, Manchester, UK; 3 School of Dentistry, Wellcome Trust Brenner Building, University of Leeds, Leeds, UK; 4 Academic Department of Trauma and Orthopaedic Surgery, School of Medicine, University of Leeds, Leeds, UK

**Keywords:** Biomaterials, Materials for devices, Nanoscale materials, Nanobiotechnology, Characterization and analytical techniques, Disease prevention

## Abstract

Bone healing is a complex process, and if not managed successfully, it can lead to non-union, metal-work failure, bacterial infections, physical and psychological patient impairment. Due to the growing urgency to minimise antibiotic dependency, alternative treatment strategies, including the use of nanoparticles, have attracted significant attention. In the present study, cerium oxide nanoparticles (Ce^4+^, Ce^3+^) have been selected due to their unique antibacterial redox capability. We found the processing routes affected the agglomeration tendency, particle size distribution, antibacterial potential, and ratio of Ce^3+^:Ce^4+^ valence states of the cerium oxide nanoparticles. The antibacterial efficacy of the nanoparticles in the concentration range of 50–200 µg/ml is demonstrated against *Escherichia coli*, *Staphylococcus epidermis,* and *Pseudomonas aeruginosa* by determining the half-maximal inhibitory concentration (IC_50_). Cerium oxide nanoparticles containing a more significant amount of Ce^3+^ ions, i.e., FRNP, exhibited 8.5 ± 1.2%, 10.5 ± 4.4%, and 13.8 ± 5.8% increased antibacterial efficacy compared with nanoparticles consisting mainly of Ce^4+^ ions, i.e., nanoparticles calcined at 815 °C.

## Introduction

Bone infections are frequent due to the increasing incidence of trauma and elective surgeries^1–3^. Despite all the efforts to maintain an aseptic operating environment, infectious bacteria may be present during treatment, in the air in the operating room, surgical equipment, contaminated orthopaedic/medical device, and resident microbiota already present on the patient's skin^4^. Epidemiological studies suggest that between 2 and 5%^5,6^ of all implant-related procedures are likely to be further complicated due to post-operative infections, leading to increased substantial health-related costs and prolonged hospitalisation, revision surgeries, and mortality^7–9^. The treatment of infection is complex involving parenteral or systemic drug administration and, in extreme cases, debridement of bone and tissue due to compromised blood circulation^10^. Drug administration for infection control uses a broad range of antibiotics, leading to multidrug-resistant bacterial strains^8,11^. As a result, the treatment of infectious diseases requires higher and prolonged dosages of multiple antibiotics, often leading to intolerable toxicity.

Biofilm refers to a well-organised community of bacteria surrounded by a matrix for protection that usually adheres to a surface/implant within the human body. Bacteria being in a planktonic state enable a change in the gene expression pattern. The genes responsible for producing bacterial extracellular polymeric substances (EPS) are activated and expressed^12^. The excretion of EPS further facilitates the exponential growth of bacteria leading to biofilm formation^7,13,14^. The presence of a biofilm hinders the host's immune response and antibiotic delivery; hence, biofilms are one of the major causes of bacterial resistance development^8^. Due to the growing urgency to minimise antibiotic dependency, alternative treatment strategies, including the use of nanoparticles, have attracted significant attention as these types of particles manifest unique physicochemical properties. The unique properties are only apparent on the nanoscale, e.g., surface area to volume ratio, surface charge, and valence state compared with bulk counterparts^15^.

Emerging trends in antibacterial control are to deliver nanoparticles at the site and control bacterial proliferation by stopping the enzyme and gene expression. Several types of inorganic nanoparticles (ZnO, TiO_2_, Ag) emerge as novel antibacterial agents and have proven their effectiveness in treating infectious diseases^16^. The antibacterial efficacy of nanoparticles is affected by the size, shape, surface charge, and surface area to volume ratio^15^. Nanoparticles size is essential with regards to biological functions as the morphological dimensions are comparable with (i) small biological molecules (1–10 nm), (ii) viruses (10–100 nm), and (iii) the ability to attack biological entities without changing their functions^15^. The antibacterial mechanism is likely to be attributed to  the nanoparticles' ability to enter biofilms; unlike antibiotics, nanoparticles directly attack the cell wall of bacteria by attaching via (i) electrostatic interaction, (ii) Van der Waals forces as well as (iii) receptor-ligand and hydrophobic interactions^8^ disrupting the integrity of the bacterial cell wall leading to cell death^17^.

## Ceramic nanoparticles for antibacterial properties

Nanoparticles can prevent bacteria from mutating via cell death, replacing or reducing conventional antibiotics^18^. The antibacterial properties of metal and metal oxide nanoparticles such as silver^19^, copper^20^, zinc oxide^21^, and titanium dioxide^22^ are known to alter the metabolic activity of Gram-positive and Gram-negative bacteria^8^. Zinc oxide nanoparticles are found to inhibit *Staphylococcus aureus,* whereas concentration-dependent silver nanoparticles exhibited antimicrobial activity against *Escherichia coli* and *Pseudomonas aeruginosa*^23^. However, specific nanoparticles, i.e. silver, are toxic to host cells even at low doses despite exhibiting antibacterial properties^24^. Additionally, triangular-shaped silver particles exhibited higher antibacterial properties than spherical or rod-shaped nanoparticles^25^. Nano-silver intraperitoneal injection and its dispersion through blood adversely affect the lungs, liver, gastroenterological tract and brain tissues^26^. For this reason, the use of silver in treating internal infections is now severely curtailed. Other studies have reported gold^27^, magnesium oxide^28^ and copper oxide^8,29,30^ based nanoparticles prevented the formation of biofilm, which is linked to the high surface area-to-mass ratio, i.e., smaller sized less than 10 nm^7,8^.

## Cerium oxide particles and processes

Cerium oxide nanoparticles have attracted a great deal of interest as antibacterial agents due to the ability to cycle between the two valences states (Ce^3+^ and Ce^4+^), leading to the formation of oxygen vacancies in the lattice. The apparent beneficial oxygen buffering capability enables the nanoparticles to act as a catalyst for oxidation and reduction reactions^31^, manifesting a unique antibacterial mechanism^18^. The intrinsic bivalence of cerium oxide nanoparticles induces antioxidant capabilities^32^ (catalytic oxidation and reduction), protecting the cells from oxidative stress, inflammation^33^, and potential radiation damage^34^. Nanoscale cerium oxide can mimic an antioxidant enzyme superoxide dismutase found in all living cells^35^. Superoxide dismutase, catalase and glutathione are considered the body's cellular defence as they catalyse the breakdown of potentially harmful oxygen molecules known as reactive oxygen species (ROS)/free radicals, thus, preventing tissue damage within the body. The primary role of antioxidants is to reduce excessive amounts of ROS/free radicals, hence combating oxidative stress-related diseases^36^. Nanoscale cerium oxide presents relatively low or no toxicity to mammalian cells^37–40^ and is proven to decrease catalysts of chronic inflammation via nanotherapeutics^33^, as well as demonstrating the ability to enhance neuroprotection^41^.

Cerium oxide nanoparticles exhibit pro-oxidative behaviour, depending on the environment, i.e., oxidative stress is induced directed at bacteria^42^. Conversely, several studies conclude no apparent antibacterial effect of cerium oxide nanoparticles^43,44^. However, other findings highlight possible adverse effects of the cerium oxide nanoparticles, where oxidative stress was induced in epithelial human lung cells^45^. The range of conflicting data in the literature may be attributed to varying manufacturing processes, chemical solvents not entirely removed, irregular pH during production and increased calcination temperatures^46^. The redox properties of cerium oxide nanoparticles can be tuned via materials preparation method, drying method, particle size, surface chemistry, particle shape and level of dopant materials^31^. The drying method of nanoparticles is a vital aspect to consider as nanoparticles tend to agglomerate, which adversely affects the physicochemical properties of the particles^47^. Thus, the procedures employed to evaluate the antimicrobial and antibacterial properties associated with cerium oxide nanoparticles may also make it challenging to form significant conclusions.

## Crystalline defect structure

In the fluorite structure of cerium oxide, the redox equilibrium between the two valence states (Ce^3+^:Ce^4+^) may be explained by considering the reaction in the presence of oxygen gas, as shown in Eq. (1):

1 $$2{{CeO}}_{2} { } \left( s \right) = {{ C}}e_{2} {{O}}_{3} \left( {{s}} \right) + \frac{1}{2} {{O}}_{2} \left( {{g}} \right)$$

**Figure 1:**
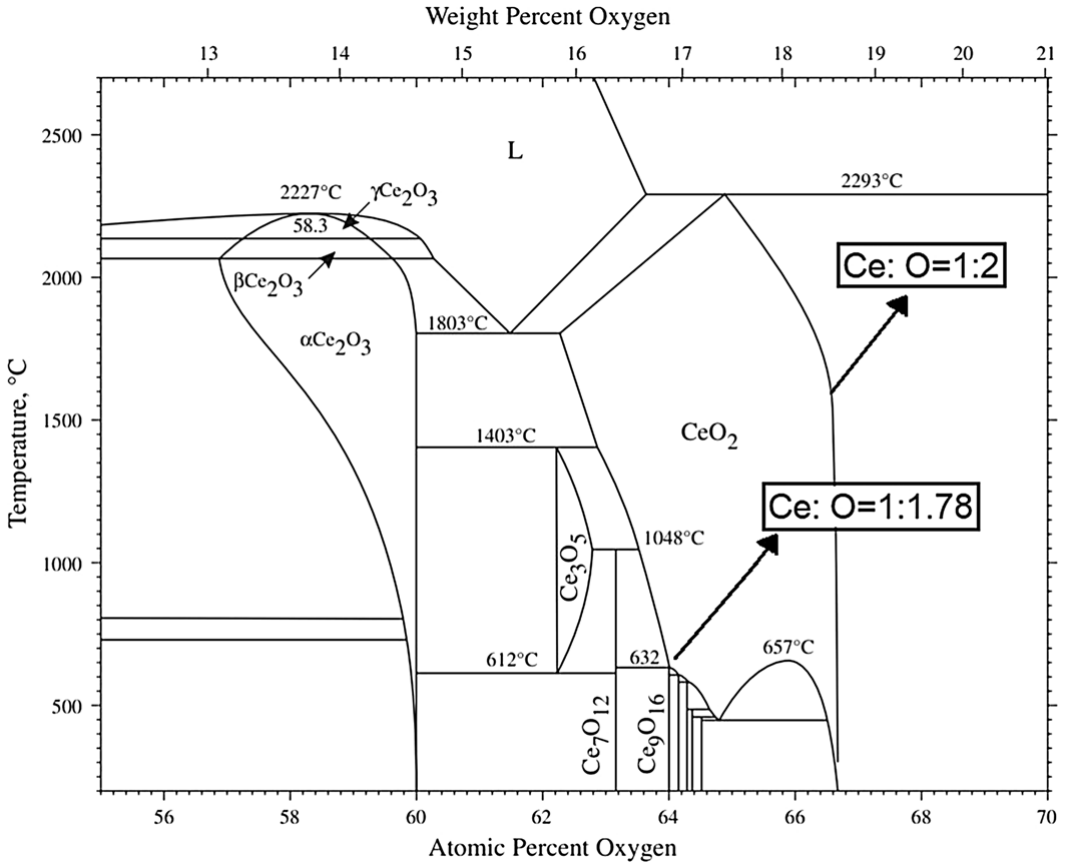
The phase diagram of Cerium-Oxygen system showing non-stoichiometric cerium adapted from H. Okamoto, *Journal of Phase Equilibria and Diffusion* volume 29, pages 545–547 (2008)^49^.

The Ce–O phase diagram shown in Fig. 1 highlights the solid solution homogeneity region for CeO_2_ whilst also confirming the presence of Ce_2_O_3_ as a separate compound. CeO_2_ can be considered in terms of oxygen vacancies where the intrinsic presence of oxygen vacancies in the CeO_2_ crystal structure renders CeO_2_ into a CeO_2-x_ non-stoichiometric oxide with '*x*' vacant oxygen sites^48^. The ratio of Ce^3+^:Ce^4+^ ionic states compensate against vacant oxygen sites and may be represented by the following equation:

2 $${{CeO}}_{2} \left( {{s}} \right) = {{CeO}}_{2 - x} \left( {{s}} \right) = {{Ce}}_{1 - x}^{4 + } {{Ce}}_{x}^{3 + } {{O}}_{2 - 0.5x}^{2 - } \left( {{s}} \right) = {{Ce}}_{1 - x}^{4 + } {{Ce}}_{x}^{3 + } {{O}}_{2 - 0.5x}^{2 - } \blacksquare_{x} \left( {{s}} \right)$$

In Eq. (2), the oxygen vacancy is shown by $\blacksquare_{x}$ where *x* is the fractional value of vacant sites in the fluorite structure. The oxygen vacancies in the CeO_2-0.5×_ fluorite are dependent on the oxygen partial pressure and temperature during heat treatment; thus, they are likely to determine the equilibrium ratios of the two valence states, expressed in Eqs. (1) and (2).

In the present work, cerium oxide nanoparticles (Ce^4+^:Ce^3+^) have been selected for detailed investigation of synthesis, structural spectroscopic analysis, and antibacterial properties. We have investigated and characterised the properties of three types of nanoscale cerium oxides, i.e., commercial nanoparticles (RNP4), freeze-dried (FRNP), and calcined (C280, C385 and C815). The analysis of bacterial growth in the presence of synthesised bivalent cerium oxide nanoparticles has also been explored to investigate the antibacterial efficacy of cerium oxide nanoparticles with varying Ce^4+^:Ce^3+^ ratios. Common Gram-negative and Gram-positive bacteria associated with orthopaedic infections, i.e. *E. coli* (−ve)^50,51^, *P. aeruginosa* (−ve)^52,53^ and *S. epidermis* (+ve)^4,50,54^ were selected. The half-maximal inhibitory concentrations (IC_50_) were determined, corresponding to reducing bacterial growth by 50%.

## Experimental

### Reagents and materials

Reagents and materials used for the synthesis of cerium oxide nanoparticles were of analytical grade (99.99% pure), i.e., cerium nitrate hexahydrate (Ce(NO_3_)_3_·6H_2_O) [Sigma-Aldrich, CAS: 10294-41-4], cerium (IV) oxide (CeO_2_) [Sigma-Aldrich, CAS: 1306-38-3], cerium (III) phosphate (CePO_4_) [Alfa Aesar, CAS: 13454-71-2], and sodium hydroxide (NaOH) [Sigma-Aldrich, CAS: 1310-73-2].

### Material synthesis

#### Freeze-dried cerium oxide nanoparticles

The nanoparticles were synthesised via a hydroxide mediated method where Ce(NO_3_)_3_·6H_2_O was used as a precursor, as described below in reaction steps 3–5. Hydroxide medicated approach was chosen based on the simplicity, reproducibility, and ability to synthesise spherical nanoparticles. As demonstrated by our results, the hydroxide synthesis route also offers better control over particle size and valence state distribution, which is essential for controlling the antibacterial properties reported. Briefly, 10.85 g Ce(NO_3_)_3_·6H_2_O(s) was dissolved in 250 ml distilled water under continuous stirring for 20 min resulting in a 0.1 M solution (A). Then 0.3 M NaOH solution was added dropwise under continuous stirring to the solution (A) at 50 °C for promoting hydrolysis of cerium oxide nanoparticles summarised via Eqs. (3)–(5). The solution was covered with aluminium foil and left at 50 °C under continuous stirring for 24 h. The nanoparticles were filtered and washed five times with distilled water and ethanol. The collected nanoparticles were frozen at –80 °C for 24 h and then placed into a freeze drier set at −100 °C and pressure of 43 mTorr for 24 h.**Synthesis reaction**

3 $${\text{Ce (NO}}_{{3 }} {)}_{{3}} {\text{ 6 H}}_{{2}} {\text{O + 3 NaOH }} \to {\text{ Ce(OH)}}_{{3}} {\text{ + 3 Na (NO}}_{{3}} {\text{) + 6 H}}_{{2}} {\text{O}}$$

**Precipitation**

4 $${\text{Ce}}^{{3 + }} {\text{ + 3 OH}}^{ - } { } \to {\text{ Ce (OH)}}_{{\text{3 (s)}}}$$

**Oxidation**

5 $${\text{4 Ce}}^{{3 + }} {\text{ + 12 OH}}^{ - } {\text{ + O}}_{{2}} \to {\text{ 4 CeO}}_{{{2 }\left( {\text{s}} \right) }} {\text{ + 6 H}}_{{2}} {\text{O}}$$

#### Furnace dried cerium oxide nanoparticles

The hydroxide mediated synthesised cerium oxide nanoparticles were placed into a furnace at 80 °C for 24 h for drying. Thermal analysis of the furnace dried powder was used to determine the optimal calcination temperature. All the synthesised cerium oxide nanoparticles samples are presented in Table 1 with sample code names, corresponding formulas, and synthesis method.

**Table 1:** Summary of commercial and synthesised nanoparticles with the post-synthesis cryogenic and thermal treatments.

Code	Description	Chemical formula	Synthesis
RNP4	Cerium oxide	CeO_2_	Sigma-Aldrich, CAS: 1306-38-3
FRNP	Freeze dried	–	Hydroxide mediated approach then freeze-dried
FUNP	Furnace dried	–	Hydroxide mediated approach then dried @ 80 °C
C280	Calcined @ 280 °C	–	Heat treatment of FUNP (280 °C, 2 h)
C385	Calcined @ 385 °C	–	Heat treatment of FUNP (385 °C, 2 h)
C815	Calcined @ 815 °C	–	Heat treatment of FUNP (815 °C, 2 h)

### Characterisation

#### Thermal analysis

The Perkin Elmer STA 8000, used to study the phase transformation and chemical reactions, covered the temperature heating range from 30 to 1000 °C. The thermal analysis characterisation of furnace dried ceria was essential for optimising the calcination process without promoting the growth of nanoparticles. The isochronal heating rate of 20 °C min^−1^ was used to determine the optimal calcination temperature for comparative studies for the furnace dried cerium oxide nanoparticles samples.

#### Fourier transform infrared (FTIR) and ultraviolet–visible (UV–Vis) spectroscopy

The molecular vibration spectroscopic analysis of synthesised powders (FUNP, FRNP, C285, C385 and C815) was analysed and characterised using the attenuated total reflection (ATR) mode in the Vertex 70 FTIR spectrometer. The beam splitter was KBr, and the light source used was a MIR lamp. Each sample was scanned 32 times in the 400–4000 cm^−1^ range. The spectral resolution was 4 cm^−1^. For the characterisation of the electronic absorption spectra of the nanoparticles, the PerkinElmer^®^, LAMDA 950 UV/VIS/NIR spectrometer was used. A homogeneous clear suspension of nanoparticles in deionised water at concentrations of 0.5 mg/ml was used to collect the absorption spectrum between 190 and 500 nm; in this wavelength range, the valence change due to the electronic states of cerium oxide is observed.

#### X-ray powder diffraction (XRPD)

A D8 X-ray powder diffractometer using the *K*_α_ radiation of Cu (λ = 0.15406 nm) was used to determine the crystalline structure of all the samples of synthesised nanoparticles. For powder diffraction, the samples were analysed in the Bragg angle (2*θ*) scanning range of 10°–80° at a scan speed of 5 s with a step size of 0.03°. The recorded patterns were analysed using the HighScore Plus software, and the Rietveld refinement was employed for peak shape and intensity analysis for ascertaining the crystallinity of mineral samples.

#### Transmission electron microscopy (TEM)

For TEM analysis, the samples were prepared by ultrasonic dispersion of the nanoparticles in methanol, after which several drops were placed onto holey carbon copper TEM grids. The particles suspended in methanol were then allowed to dry using a heat lamp. The Titan Themis Cubed 300 TEM operated at 300 kV with high brightness X-FEG and Supertwin objective lens was used for the characterisation of the nanoscale size distribution and morphological analysis. The Bright field TEM images were collected using the Gatan OneView 16 Megapixel CMOS digital camera. The selected area electron diffraction (SAED) patterns and the low magnification and dark-field (DF) TEM images were obtained to analyse the synthesised ceria's crystallinity. The electron energy loss spectroscopy (EELs) using the Gatan GIF Quantum ER imaging filter at low and high energy loss spectra were collected for characterising the coexistence of the two valence states in the calcined nanoparticles.

#### Surface area

Micromeritics Tristar 3000 was used to characterise the Brunauer–Emmett–Teller (BET) surface area of the synthesised nanoparticles using the nitrogen absorption method in the powder bed. The nanoparticles were placed into glass sample tubes, then placed into the FlowPrep™ 060. The powder samples were degassed with nitrogen and heated at 50 °C for 30 min to remove surface contaminants, i.e., water and absorbed gas.

### Bacterial culture and antibacterial properties

The nanoparticles tested during the bacterial experiments were sterilised in an autoclave by suspending the particles in a brain–heart infusion (BHI) broth, which, when cooled, were used within the hour. Optical density and viable count (in colony-forming units, CFUs) measurements were carried out to assess the antibacterial characteristics of the nanoparticles over 48 h. An initial optical density OD_600_ of 0.015 was selected based on the literature^55^ and was kept constant for all experiments to ensure reproducibility. Triplicates of bacterial experiments were conducted, where the data collected underwent statistical analysis and is displayed as mean ± standard deviation (SD). Significant statistical differences were analysed using a one-way analysis of variance (ANOVA) between the average of two or more results. Values of *p* < 0.05 were considered statistically significant.

#### Brain heart infusion agar plates and broth

Brain–heart infusion (BHI) agar [Sigma Aldrich, BHI Agar #70138] plates [Sigma-Aldrich, BRAND^®^ disposable petri dish with lid #BR452005] were prepared following the manufacturer's protocol. The solution was sterilised by autoclaving at 121 °C for 15 min. Once the agar solution had cooled, it was poured into a set of sterile agar plates under aseptic conditions and left to solidify. The agar plates were then stored at < 4 °C until they were required. The BHI broth [Sigma Aldrich, BHI Broth #53286] was prepared by dissolving 37 g of broth powder in 1 L of distilled water. The broth was autoclaved at 121 °C for 15 min to sterilise and refrigerated at < 4 °C for future experiments.

#### Growth of bacterial strains

Bacterial stock cultures of *E. coli*, *S. epidermis* and *P. aeruginosa* were procured from a stock of 30% glycerol solutions kept at –80 °C. Ten µl sterile loops were used to streak *S. epidermis*, *P. aeruginosa* and *E. coli* onto Brain Heart Infusion (BHI) agar plates. Inoculated plates were incubated at 37 °C for 24 h, after which a single colony was picked from each bacterium type and grown in 25 ml of BHI broth in an incubator at 37 °C 150 rpm for 24 h. This process enabled the production of fresh bacterial suspension for further use by inoculation.

#### Optical density measurements without and with nanoparticles

Optical density (OD) measurement is a widely used method to assess the number of growing bacteria in a culture; thus, the absorbance values of bacterial suspensions can be measured using a photometer^56^. The initial optical densities of each bacterium type were measured using the Jenway 6305 UV/Visible Spectrophotometer at 600 nm (OD_600_). The bacterium suspensions were diluted to an OD_600_ of 0.015^55,57^ using BHI broth to ensure reproducibility. Triplicate bacterial solutions for each bacterium were produced, and the growth rate without the addition of the nanoparticles was measured at 2, 4, 24 and 48 h. To investigate the antibacterial properties of selected nanoparticles (FRNP, C385 and C815), various concentrations (50, 100, and 200 µg/ml) of sterilised nanoparticles suspended in BHI broth were added to the bacterial suspensions OD_600_ of 0.015. The OD_600_ was measured after 2, 4, 24, and 48 h and compared with the optical density measurements of bacterial suspensions containing no nanoparticles. The average values ± SD are reported.

## Results of materials characterisations

### Simultaneous thermal analysis

**Figure 2:**
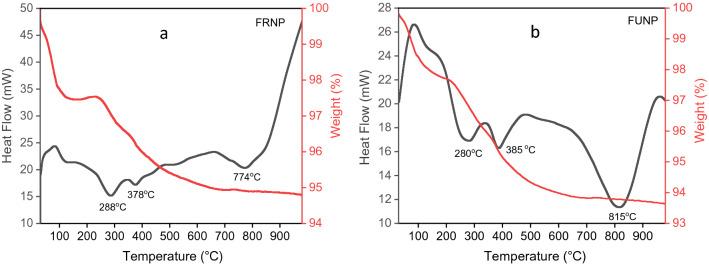
Simultaneous thermal analysis of synthesised cerium oxide nanoparticles, (**a**) FRNP nanoparticles and (**b**) FUNP nanoparticles, the endothermic peaks signify potential structural changes and a change in the Ce^3+^:Ce^4+^ ratio. The heating temperature range was 30–1000 °C at a heating rate of 20 °C/min.

When nanoscale particles are heat-treated, the temperature plays a significant role in determining the structural and chemical changes, e.g., higher calcination temperatures often lead to larger nanoparticle sizes^58^ which affects the Ce^3+^:Ce^4+^ ratio. The simultaneous thermal analysis results for the synthesised FRNP and FUNP cerium oxide nanoparticles are displayed in Fig. 2. The results indicate a two-step weight loss process occurring in both types of nanoparticles up to 980 °C. The initial weight loss of ~ 2.3% is observed between 30 and ~ 280 °C for both the FRNP and FUNP nanoparticles, corresponding to the removal of adsorbed water in the crystalline structure of cerium oxide nanoparticles^58^. The second weight losses for FRNP and FUNP nanoparticles are 2.9% (between ~ 280 and 774 °C) and 4.1% (between ~ 280 and 815 °C).

The peaks observed in FRNP and FUNP nanoparticles are endothermic and occur at different temperatures, likely due to processing conditions. The total weight loss in FRNP is ~ 5.3% against the ~ 6.4% value for FUNP. The thermal data for both types of nanoparticles display changes in the baseline, depending on the phase transition condition. The first two peaks in FRNP at 288 and 378 °C are comparable with the peaks in FUNP at 280 and 385 °C, respectively. The shape of the two endothermic peaks are associated with the rate of water evaporation (free water, water of crystallisation and OH^−^). The data suggests that the overall phase change reactions in these two types of ceria nanoparticles are different. Figure 2a shows two high-temperature phase transformation reactions, one above 500 °C, and the second above 675 °C, with a peak at 774 °C. The overall rate of weight loss at the ~ 500 °C reference point in the FRNP is much lower than that in the FUNP ceria.

Compared to FUNP, the 500 °C peak is barely discernible due to the magnitude of the ensuing phase transformation, which peaks at 815 °C. Since the shape of an endothermic peak in STA entails the rate of completion of a corresponding phase transformation, it may be possible to conclude semi-quantitatively that the thermal behaviour and corresponding phase transformation in FRNP and FUNP differ significantly. The phase transformation above 650 °C in both types of nanoparticles is associated with the reactions exhibited in Eqs. (1) and (2). Above 500 °C, the rate of weight loss is prolonged, especially for the FRNP nanoparticles. The endothermic peak temperatures at (i) 280 °C, (ii) 385 °C and (iii) 815 °C for FUNP nanoparticles were selected to investigate the structural changes further and determine the Ce ^3+^:Ce^4+^ ratios.

### X-ray diffraction analysis

**Figure 3:**
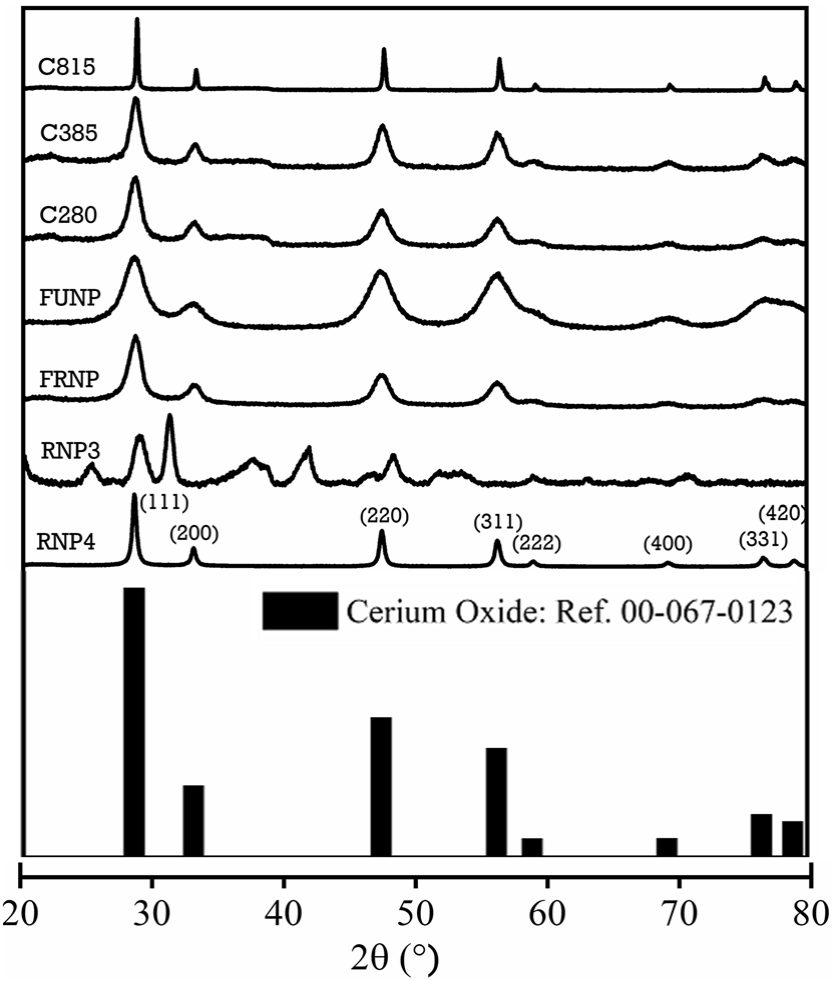
Comparing the X-ray powder diffraction peak broadening in commercial and synthesised cerium oxide nanoparticles calcined at 280 °C, 385 °C and 815 °C. The 2θ scanning range was 20°–80° at a scan speed of 5 s and increment of 0.03°. The obtained data were compared to the reference pattern of cerium oxide JCPDS 00-067-0123.

The XRD diffraction spectra (Fig. 3) for the synthesised nanoparticles exhibit eight major characteristic peaks observed at the 2θ values, 28.49°, 33.00°, 47.38°, 56.12°, 58.96°, 69.45°, 76.51° and 78.89°, which correspond to (111), (200), (220), (311), (222), (400), (331) and (420) Miller indices, respectively. The synthesised nanoparticles are single phased material, as no secondary peaks associated with Ce(OH)_3_ and Na(NO_3_) are observed. The X-ray powder diffraction profiles of FRNP and FUNP nanoparticles depict broader peaks than the commercial powder RNP4. On the other hand, the full width half maximum (FWHM) for heat-treated nanoparticles became narrower with increasing calcination temperatures, e.g., 280 °C to 815 °C. The sharp XRD peaks signify an increase in the crystallinity of calcined cerium oxide nanoparticles^59^.

The cerium oxide nanoparticles analysed crystalline structures agree well with the fluorite phase CeO_2_ [JCPDS 00-067-0123 reference] with a lattice parameter of *a* = 5.423 Å, and space group *Fm3m*. The average crystallite size ranges were calculated using the Braggs law (*n λ* = *2 d sin θ*), and the Debye–Scherrer formula given below:

6 $$D = 0.9 \, \lambda {/}\beta \cos \, \left( \theta \right)$$

D is the average crystallite size, λ is the X-ray wavelength, *β* is the FWHM of the Bragg reflection in radians, and θ is the half of the Bragg angle (2θ). The face-centred cubic (FCC) lattice parameters' *a*' were calculated from the formula, *d*_*hkl*_ = *a*/[*h*^^2^ + *k*^2^ + *l*^2^]^1/2^, where *d*_*hkl*_ corresponds to the interplanar spacing with Miller indices *h*, *k* and *l*. Table 2 displays the results of the average size range for the cerium oxide nanoparticles investigated after Rietveld refinement. The values of the calculated lattice parameters decrease with the increasing calcination temperature, which increases the average crystallite size of cerium oxide. The room temperature values of the lattice parameters of cerium oxide in RNP4, FRNP and FUNP nanoparticles are larger in dimension than the calcined nanoparticles (C280, C385 and C185). The apparent reduction in the lattice dimensions for calcined nanoparticles may be attributed to reduced lattice defects and oxygen vacancies^60^.

**Table 2:** A comparison of the lattice dimensions, average crystallite sizes, BET surface area and pore volumes of the synthesised cerium oxide nanoparticles.

Sample	Lattice cell parameter (Å)	Crystallite size (nm)	BET surface area (m^2^ g^−1^)	BJH cumulative pore volume (cm^3^ g^−1^)
RNP4	5.41	21.56 ± 6.47	36.72 ± 0.14	0.170
FRNP	5.42	4.29 ± 1.29	71.63 ± 0.47	0.167
FUNP	5.41	6.59 ± 1.97	36.40 ± 0.15	0.153
C280	5.40	6.70 ± 2.01	35.40 ± 0.11	0.162
C385	5.39	7.87 ± 2.37	46.37 ± 0.15	0.179
C815	5.37	32.59 ± 9.78	4.27 ± 0.04	0.028

### Brunauer–Emmett–Teller (BET) surface area

**Figure 4:**
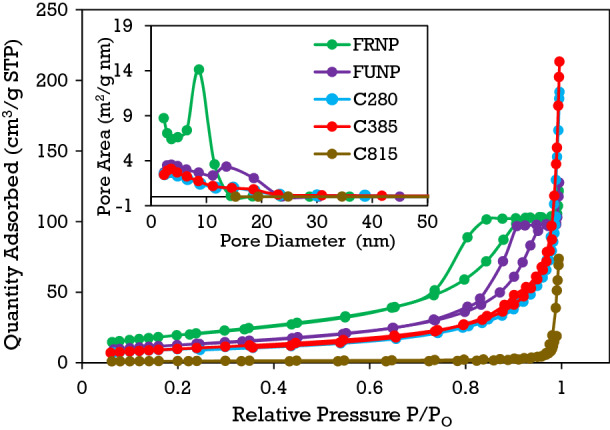
The dependence of the pore size distribution of processed cerium oxide nanoparticles using N_2_ adsorption–desorption isotherms measurements. The inset plot compares the BJH pore diameter distribution for the synthesised cerium oxide nanoparticles.

The surface area of the cerium oxide nanoparticles was determined by the physical adsorption of inert gas nitrogen. At a cryogenic temperature of 78 k (− 195 °C), nitrogen gas forms an adsorbed monolayer on the surfaces of the nanoparticles. Therefore, the adsorbed gas monolayer's measurement as a function of relative pressures yields specific surface area and surface porosity. The results of the BET surface area measurements displayed in Table 2 decrease with increasing calcination temperature, suggesting that the average size range of the calcined cerium oxide nanoparticles increases with increasing temperature, as confirmed from the comparison microscopic analysis of the TEM images in Fig. 6. Nanoparticles exhibit increased surface area compared to their bulk counterparts; the size of nanoparticles is inversely proportional to the surface area; hence, smaller nanoparticles generally exhibit a large surface area. It is evident from the TEM analysis that there is a particle size range variation for the RNP4 nanoparticles and that this variation is much more considerable than what is observed in the FRNP and FUNP samples. Additionally, agglomeration of the nanoparticles was observed for the FRNP, FUNP, C280, and the C385, which are indiscernible because the molecular nitrogen (N_2_) gas with 364 pm was used for the BET surface area measurements. A monatomic helium gas might be able to discern the differences in surface area due to nanoparticle agglomeration. Figure 4 presents the nitrogen adsorption–desorption isotherms, and the corresponding Barrett-Joyner-Halenda (BJH) pore size distributions for the synthesised cerium oxide nanoparticles. Nanoparticles exhibiting pore diameters in the 2–50 nm range are considered mesoporous in nature^61^. The adsorption–desorption isotherms illustrate type-IV isotherm curves with H3-type hysteresis loops, which are characteristics of mesoporous structured nanoparticles containing slit-like pores^62,63^. The pore diameters increase, while the BJH pore volumes decrease for nanoparticles calcined at high temperatures, as shown in Table 2.

### Fourier transform infrared and ultra-violet visible spectroscopy

**Figure 5:**
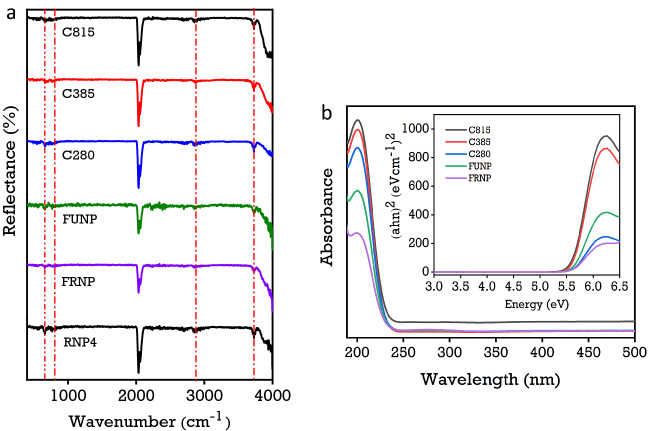
(**a**) A comparison of synthesised cerium oxide nanoparticles calcined at 280 °C, 385 °C, and 815 °C analysed using a Vertex 70 FTIR spectra from 400 to 4000 cm^−1^. The operating parameters consisted of a total of 32 scans at a resolution of 4 cm^-1^ and (**b**) UV–Vis absorbance spectra obtained from nanoparticle concentrations of 0.5 mg/ml. The inset Tauc Plot corresponds to the bandgap energies of synthesised cerium oxide nanoparticles.

The FTIR spectra of all the synthesised cerium oxide nanoparticles appear primarily similar, as shown in Fig. 5. The broad reflectance peak in the 3500–4000 cm^−1^ range is attributed to the O–H stretching associated with the hydroxyl molecules on the surface. The significant peaks at 2050 cm^−1^ are likely due to atmospheric CO_2_ and potentially adsorbed CO_2_ gas on cerium oxide nanoparticles. As shown in Fig. 5a, the intensity of surface adsorption of CO_2_ peak at 2050 cm^−1^ increases with increasing calcination temperatures. The ceria bound gas peak for the FUNP nanoparticles has a lower peak intensity than the calcined nanoparticles, i.e., C280, C385 and C815, suggesting that atmospheric CO_2_ was readily trapped during the furnace heat-treatment process. Resolving the 2050 cm^−1^ peaks in Fig. 5a highlights the doublet rotational gas phase, where the calculated peak areas increase with the temperature. The vibration bands at 500–750 cm^−1^ are assigned to the Ce–O stretching and agree with cerium oxides' vibrational assignments in the literature^40,64–66^. Electronic state absorbance spectra due to valence changes in the synthesised cerium oxide nanoparticles are shown in Fig. 5b, where the maximum absorbance peak is located at ~ 210 nm region. The electronic absorption spectrum directly represents the ratio of Ce^3+^ and Ce^4+^ ion concentrations on the surface of the nanoparticles. From the comparison of absorption data as a function of temperature, it is evident that the absorbance and the bandgap energy (eV) are strongly dependent on the heat treatment temperature. The magnitude of absorbance due to Ce^4+^ and Ce^3+^ increases with the temperature. The corresponding bandgap due to particle size reduces with increasing temperature, as shown in the Tauc plot, from ~ 5.5 to < 5.3 eV, consistent with the bandgap observed in the bulk ceria crystal.

### Transmission electron microscopy

**Figure 6:**
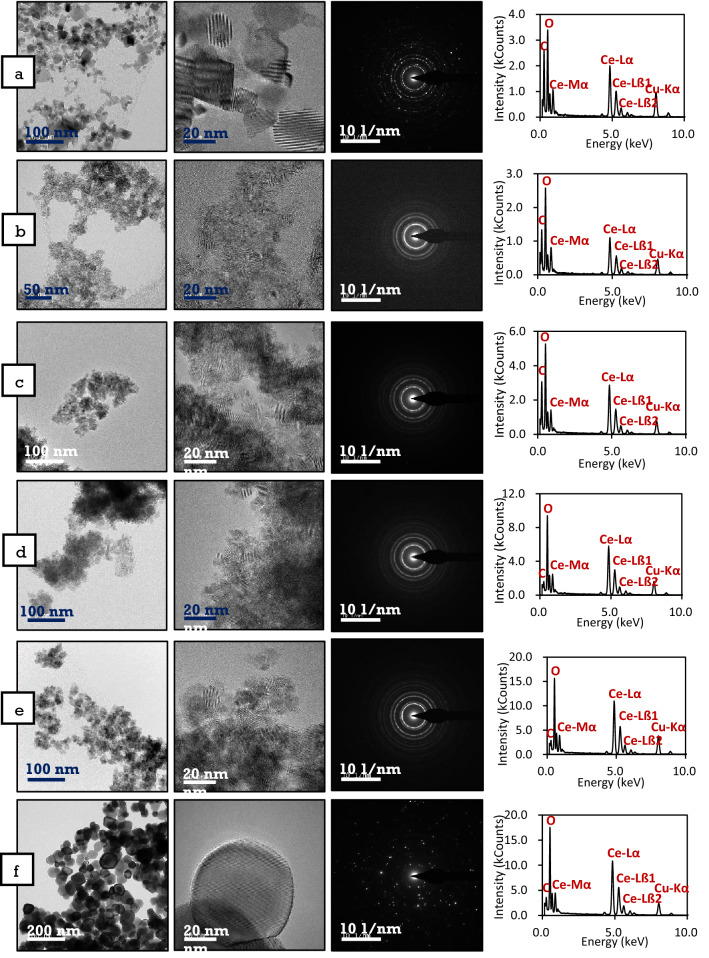
Comparison of Titan Themis Cubed 300 TEM images of nanoparticles calcined at various temperatures (**a**) RNP4, (**b**) FRNP, (**c**) FUNP, (**d**) C280, (**e**) C385, (**f**) C815, with the corresponding selected area electron diffraction patterns and energy dispersive X-ray analysis. The insets for a–f shows the EDX spectra associated with the synthesised cerium oxide nanoparticles in terms of oxygen and cerium comparison.

The majority of synthesised nanoparticles were nearly spherical, except in the case of RNP4 and C815 nanoparticles. Based on visual observation of the TEM images in Fig. 6, the size of the nanoparticles increases with increasing calcination temperatures. The increased particles size with increasing heat treatment temperatures, and agglomeration contribute to two possible mechanisms, (i) Ostwald ripening and (ii) Oriented Attachment. Ostwald ripening is temperature-dependent as it influences the interfacial surface energies and the growth rate coefficients of the nanoparticles. Larger particles are more energetically stable than smaller particles; thus, the possible dissolution of the smaller particles led to the growth of the larger sized nanoparticles. The oriented attachment mechanism is attributed to the aggregation of nanoparticles which reduces the total energy of the system and the interphase boundaries, thus leading to increase particles size^67^. The particles size for RNP4, FRNP, FUNP, C280, C385 and C815 were analysed from high-resolution TEM (HRTEM) images via ImageJ software, and the average diameter values were found to be 25, 4, 6, 8, 11 and 53 nm, respectively.

Diffraction analysis was also carried out where the three-principle low diffraction index planes for the fluorite structure in cerium oxide are (111), (110) and (100). The dominant lattice fringes observed in the HRTEM cerium oxide images correspond to the (111) planes, which is expected as the (111) plane is the closest packed plane in a face-centred cubic structure. The SAED data are therefore consistent with the X-ray powder diffraction data in Fig. 3, discussed above. The SAED patterns for all samples except RNP4 and C815 nanoparticles depict continuous ring patterns, indicating the particles' polycrystalline and nano-structural nature. By comparison, the RNP4 and C815 nanoparticles' SAED patterns depict discrete diffraction spots and spread discontinuous rings, which indicate relatively large-sized nanoparticles. The diffraction rings and reflections appear to agree with the X-ray diffraction data and confirm the fluorite structure.

### Electron energy loss spectroscopy

**Figure 7:**
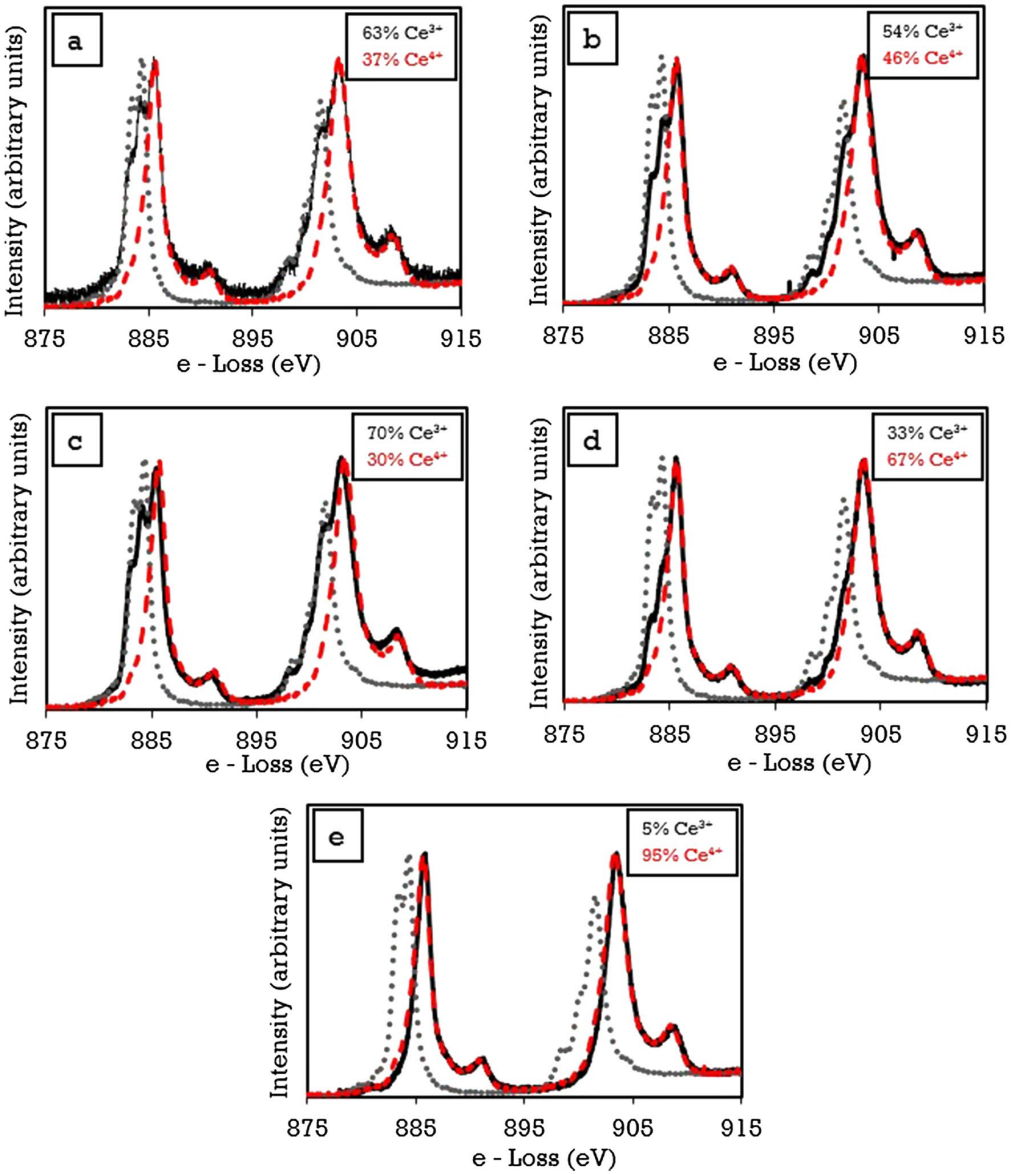
Normalized EELs spectra depicting the presence of dual valence states. (**a**) FRNP, (**b**) FUNP, (**c**) C280, (**d**) C385 and (**e**) C815. Compared with Ce^3+^ (grey) and Ce^4+^ (red) standards.

Cerium oxide is electropositive^68^ and can exist in two valence modes, i.e. Ce^3+^ and Ce^4+^. The EELs measurements were acquired by rastering the beam across several points on each nanoparticle surface. EELS spectra from two controls (Ce^3+^ and Ce^4+^) and five synthesised nanoparticles are shown in Fig. 7. In each case, the background was removed, and the Fourier-Ratio Deconvolution routines were applied prior to fitting. From the EELs spectra, the Ce M_4,5_ edges of FRNP, FUNP, C280, C385 and C815 spectra are shown where the black lines represent the sample data, the red lines represent the Ce^4+^ ion spectra, and the grey lines represent the Ce^3+^ ion spectra. Oxygen vacancies in cerium oxide form as the calcination temperature increases and the equilibrium partial are less than that from Eqs. (1) and (2). When cerium oxide nanoparticles were heat-treated in a muffle furnace in the air at an isotherm, the oxygen loss occurred and reached an equilibrium value manifested by the Ce^3+^ and Ce^4+^ ion ratio. The comparison of the Ce^3+^:Ce^4+^ ratio for the ceria particles analysed shows for larger-sized ceria particles, the ratio of Ce^3+^ and Ce^4+^ is lower than that in smaller particles. As the calcination temperature increases, the sintering tendency of particles increases, which reduces the overall surface area in contact with the surrounding atmosphere. As a result, the number of potential sites which are likely to lose oxygen reduces. However, not all the oxygen vacancies are filled, leading to a variation in the Ce^3+^:Ce^4+^ ratio. As the size of the nanoparticles increases for calcined samples, the Ce^3+^:Ce^4+^ ratio decreases as the C815 sample contains a 95% majority of Ce^4+^ ions. The presence Ce^4+^ in the FRNP, FUNP, C280 and C385 was calculated to be 37%, 46%, 30%, and 67% respectively. The obtained results confirm that the Ce^3+^:Ce^4+^ ratio is temperature-dependent. Therefore, the optimal calcination temperature must be characterised to ensure optimal Ce^3+^:Ce^4+^ to provide the highest antibacterial efficacy. Based on the EELs analysis, the FRNP, C385 and C815 nanoparticles were selected due to the particle size range and Ce^3+^:Ce^4+^ ratio to be further investigated.

## Bacterial growth

### Optical density measurements and IC_50_

**Figure 8:**
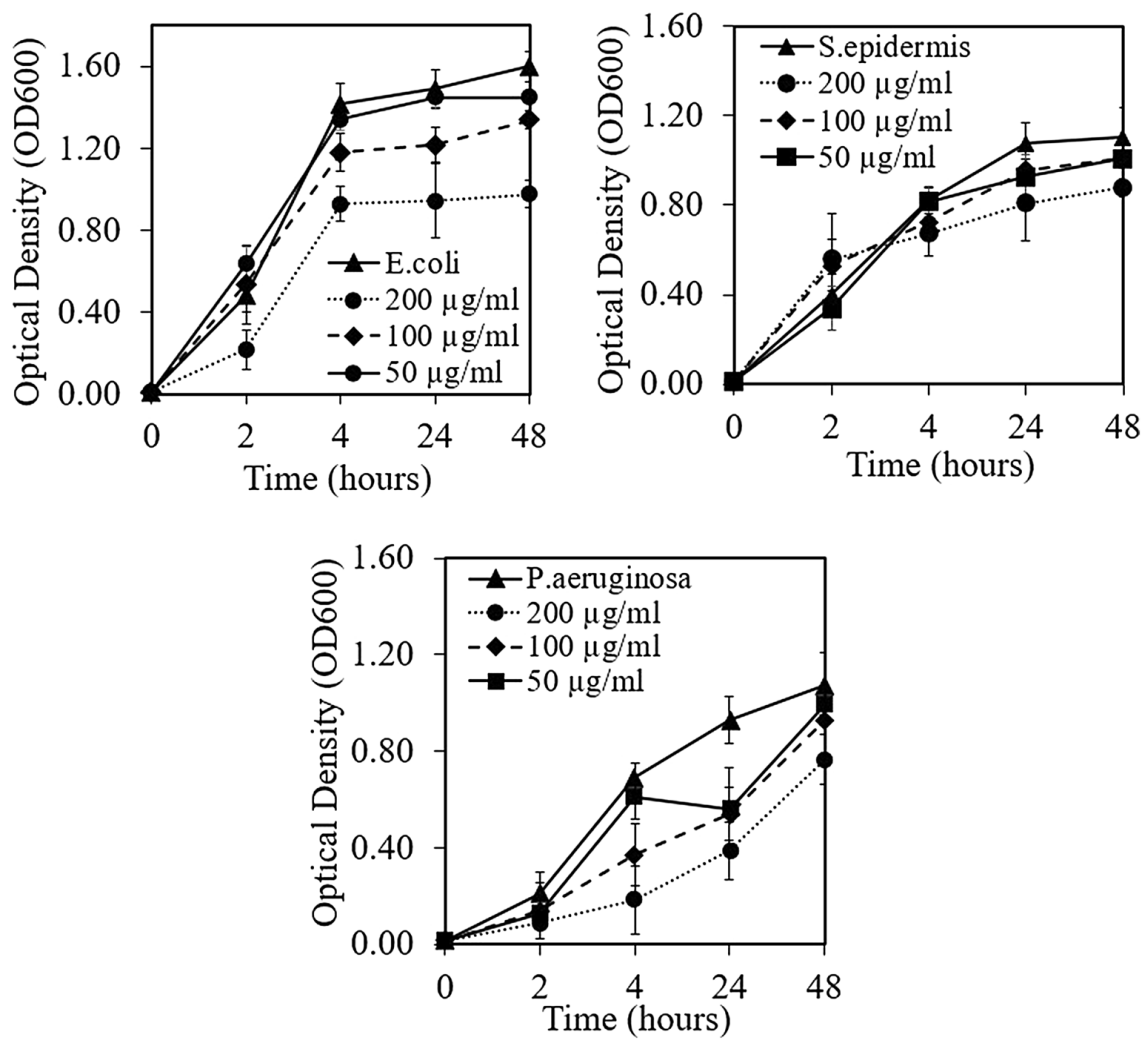
Optical density of the bacteria characterising the antibacterial properties of FRNP after direct incubation with *E. coli, P.* *aeruginosa* and *S. epidermidis* at 0, 2, 4, 24 and 48 h time points. The error bars are equivalent to the mean ± standard deviation (SD) (n = 3 in each group).

FRNP, C385 and C815 nanoparticles were selected to investigate the antibacterial properties of nanoparticles due to varying Ce^3+^:Ce^4+^ ratio and particle size distribution. All three types of nanoparticles exhibited antibacterial properties against Gram-positive and Gram-negative bacteria. However, the FRNP nanoparticles exhibited the greatest antibacterial activity, whereby a 38.8 ± 6.4% reduction was observed against *E. coli.* In contrast, a 28.7 ± 10.2% reduction was observed against *P.* *aeruginosa* after 48 h of incubation (Fig. 8). The C385 nanoparticles exhibited 33.5 ± 5.8%, 20.7 ± 8.1% and 13.9 ± 1.1% reduction of *E. coli, P.* *aeruginosa* and *Staphylococcus epidermidis*, respectively. The lowest antibacterial activity was observed for C815 nanoparticles with 20.3 ± 7.6%, 18.2 ± 5.8% and 6.4 ± 9.9% bacterial reduction expressed against *E. coli, P.* *aeruginosa* and *S. epidermidis*, respectively.

The three main antibacterial mechanisms displayed by cerium oxide nanoparticles are (a) cell wall adsorption, (b) attack proteins or cell transport and (iii) induce oxidative stress. The first antibacterial mechanism involves the positively charged CeO_2_ nanoparticles adsorbing via electrostatic interactions onto the negatively charged bacterial cell walls. The nanoparticles likely block the membrane and remain for a time impairing the viscosity of the cell wall, thus disrupting the transport exchange^69^ between the solution and the bacterial cells^17^. The second antibacterial mechanism of CeO_2_ nanoparticles has been linked to irregularly shaped nanoparticles causing damage to bacterial walls as demonstrated against Gram-positive bacteria^18,70^. The third mechanism is associated with inducing oxidative stress by generating reactive oxygen species (ROS). The ROS are developed on the bacterial cell wall surfaces from the reversible conversion of Ce^3+^ and Ce^4+^^18^. The ROS are known to attack nucleic acids, proteins and polysaccharides, causing the loss of function, thus leading to the destruction and decomposition of bacteria^71^. Based on the results obtained, the relative size of the synthesised nanoparticles is also related to antibacterial effectiveness. Similar to positively charged particles, nanoparticles with relatively large surface areas can also adsorb tightly on the negatively charged bacterial cell walls disrupting the membrane integrity, causing cell lysis^72,73^.

The half-maximal inhibitory concentrations (IC_50_) calculated from linear regression models are shown in Table 4. The FRNP nanoparticles exhibited enhanced antibacterial efficacy than the C385, and C815 nanoparticles, likely attributed to the high surface area to volume ratio and increased Ce^3+^ ions. Smaller sized cerium oxide nanoparticles appear more non-stoichiometric with respect to oxygen, which proportionately increases the number of Ce^3+^ sites for facilitating the sites for redox reactions. There is contradictory evidence in the literature regarding increased antibacterial effectiveness against Gram-positive and Gram-negative bacteria. Several studies have demonstrated superior action of cerium oxide nanoparticles against Gram-negative bacteria *E. coli* compared with Gram-positive bacteria *Bacillus subtilis*^74^. However, other studies expressed moderate antibacterial efficacy against Gram-negative bacteria, i.e., *P. aeruginosa* and *Proteus vulgaris*, with increased activity against Gram-positive bacteria *S. aureus* and *Streptococcus pneumoniae*^70,75^. The variation of the cerium oxide antibacterial properties is likely related to possible oxygen defects in the nanoparticles, variation in the bandgap energies^76^, the Ce^3+^:Ce^4+^ ratio, irregular morphologies and possibly low dispersity^77^.

**Table 4:** The half-maximal inhibitory concentration (IC_50_) values of FRNP, C385 and C815 tested with *E. coli, P.* *aeruginosa* and *S. epidermidis*.

IC_50_ (µg/ml)	*E. coli*	*P. aeruginosa*	*S. epidermis*
FRNP	340 ± 4.0	558 ± 3.9	580 ± 4.6
C385	365 ± 6.6	619 ± 5.2	741 ± 5.6
C815	563 ± 8.2	663 ± 9.4	785 ± 5.8

### Conclusions

We have successfully synthesised cerium oxide nanoparticles in this study, as confirmed by XRD, BET and TEM analysis. Two valence states were identified in FRNP, FUNP, C280, C385 and C815 nanoparticles; however, the Ce^3+^:Ce^4+^ ratio reduced with increasing calcination temperature. The nanoparticles exhibited increased antibacterial effectiveness against Gram-negative bacteria (*E. coli* and *P. aeruginosa*) compared to the Gram-positive bacteria (*S. epidermis*). The half-maximal inhibitory concentrations (IC_50_) to reduce bacterial growth by 50% were lower for FRNP than the C385 and C815 nanoparticles. Based on the findings herein, it appears that the antibacterial properties of nanoparticles are dependent upon two main factors, (i) the type of bacteria and (ii) the physicochemical properties of the nanoparticles. The development and utilisation of biomaterials consisting of Ce^3+^ and Ce^4+^ nanoparticles can provide new approaches for preventing and treating bone infections in high-risk patients (i.e., diabetics, immunocompromised) and in compromised environments predisposed to develop infections such as open fractures, avascular bone necrosis and prolonged reconstruction procedures. Moreover, such a strategy can help overcome the ever-growing concern of bone infections and overcome antibiotic-mediated resistance.

## Data Availability

The datasets used and/or analysed during the current study are available from the corresponding author on reasonable request.
